# Social Determinants of Health Screening at an Urban Emergency Department Urgent Care During COVID-19

**DOI:** 10.5811/westjem.59068

**Published:** 2023-07-17

**Authors:** Haeyeon Hong, Kalpana Narayan Shankar, Andrew Thompson, Pablo Buitron De La Vega, Rashmi Koul, Emily C. Cleveland Manchanda, Sorraya Jaiprasert, Samantha Roberts, Tyler Pina, Emily Anderson, Jessica Lin, Gabrielle A. Jacquet

**Affiliations:** * Boston Medical Center, Department of Emergency Medicine, Boston, Massachusetts; † Boston University School of Medicine, Department of Emergency Medicine, Boston, Massachusetts; ‡ Brigham and Women’s Hospital, Department of Emergency Medicine, Boston, Massachusetts; § Boston University School of Medicine, Department of Internal Medicine, Boston, Massachusetts; ¶ Boston University School of Medicine, Boston, Massachusetts; || American Medical Association, Center for Health Equity, Boston, Massachusetts

## Abstract

**Introduction:**

Social determinants of health (SDoH) impact patients’ health outcomes, yet screening methods in emergency departments (ED) are not consistent or standardized. The SDoH-related health disparities may have widened during the coronavirus 2019 (COVID-19) pandemic, especially among patients who primarily receive their medical care in EDs. We sought to identify SDoH among ED urgent care patients during the COVID-19 pandemic at an urban safety-net hospital, assess the impact of the pandemic on their SDoH, study the feasibility of SDoH screening and resource referrals, and identify preferred methods of resource referrals and barriers to accessing resources.

**Methods:**

Research assistants screened ED urgent care patients using a validated SDoH screener, inquiring about the impact of COVID-19 on their SDoH. A printed resource guide was provided. Two weeks later, a follow-up telephone survey assessed for barriers to resource connection and patients’ preferred methods for resource referrals. This study was deemed exempt by our institutional review board.

**Results:**

Of the 418 patients presented with a screener, 414 (99.0%) patients completed the screening. Of those screened, 296 (71.5%) reported at least one adverse SDoH, most commonly education (38.7%), food insecurity (35.3%), and employment (31.0%). Housing insecurity was reported by 21.0%. Over half of patients (57.0%) endorsed COVID-19 affecting their SDoH. During follow-up, 156 of 234 (67%) attempted calls were successful and 36/156 (23.1%) reported attempting to connect with a resource, with most attempts made for stable housing (11.0%) and food (7.7%). Reasons for not contacting the provided resources included lack of time (37.8%) and forgetting to do so (26.3%). Patients preferred resource guides to be printed (34.0%) and sent via text message to their mobile devices (25.6%).

**Conclusion:**

Many urgent care patients of this urban ED reported at least one adverse SDoH, the majority of which were exacerbated by the COVID-19 pandemic. This finding further emphasizes the need to allocate more resources to standardize and expand SDoH screening in EDs. Additionally, hospitals should increase availability of printed or electronic SDoH resource guides, resource navigators, and interpreters both during and after ED visits.

## INTRODUCTION

### Background

Social determinants of health (SDoH) impact patients’ health outcomes,^1^,^2^ yet attempts to capture this information in the emergency department (ED) have not been consistent or standardized.^3^–^5^ Prior research confirms that the ED serves a particularly vulnerable population with high rates of social needs.^6^ By law, emergency physicians are mandated to care for every patient who seeks care from the ED, whether for medical or social needs, further necessitating better understanding of patients’ SDoH.^7^,^8^ However, most EDs currently screen for SDoH at a much lower rate than for other social risks including violence, substance use, or mental health.^4^ In 2017, an electronic health record (EHR)-based SDoH screening and referral program was developed at our urban, safety-net hospital for the adult ambulatory care clinics.^9^ This program validated a screener assessing eight SDoH domains: housing; food; transportation; utilities; employment; medication; dependent care; and education. A partial SDoH screening implemented in 2019 in the adult ED at our institution was limited to patients covered by Medicaid and those who were uninsured. With these criteria only a small fraction of total ED patients was screened for SDOH, thereby missing many safety-net hospital patients facing tenuous social circumstances.

### Importance

Although the ED cares for many patients with significant social needs negatively affecting their health outcomes, little is known about the prevalence of unmet social needs of this patient population. Additionally, the coronavirus disease 2019 (COVID-19) pandemic has penetrated every aspect of daily life and may disproportionately affect the SDoH of the patients cared for at safety-net hospitals.^10^

### Goals of this Investigation

In this study we aimed to understand the burden of SDoH among ED urgent care patients during the pandemic and evaluate the feasibility of implementing a SDoH screening and a standard referral guide provision in the ED urgent care setting.

## METHODS

### Study Design and Setting

This observational study assessed the number of patients who screened positive for adverse SDoH. For patients who endorsed at least one adverse SDoH, the study assessed the impact of COVID-19 on SDoH. We evaluated the feasibility of screening for SDO0H in ED urgent care by determining the proportion of patients who agreed to participate and completed the screening process. The demographics of the participating urgent care patients were compared to those of the general adult ED patients. This study was deemed exempt by the our institutional review board.

### Selection of Participants

A convenience sample was taken by trained research assistants (RA) who approached all patients ≥18 years old in the ED urgent care for 3–4 hours between the peak hours of 8 am–4 pm on weekdays. Participants were excluded if they were experiencing altered mental status, had been screened within the last six months, or if screening would interfere with necessary medical care. Patients with limited English proficiency were screened using a professional telephone interpreter.

**Population Health Research Capsule**

What do we already know about this issue?

*Social determinants of health (SDoH) impact patients’ health outcomes, especially with the widened disparities during the COVID-19 pandemic*.

What was the research question?

*We identified SDoH among a cohort of patients in a safety-net urgent care and the feasibility of SDoH screening and referrals*.

What was the major finding of the study?

*Screening feasibility was 99%, and 71.5% reported at least one SDoH, most commonly education (38.7%) and food (35.3%)*.

How does this improve population health?

*More resources need to be allocated to standardize and expand SDoH screening in the ED and to further optimize the social resource provision process*.

### Screening, Referral, and Assessments

Patients who agreed to participate were screened for eight SDoH domains: housing; food; transportation; utilities; employment; medication; dependent care; and education. For clarification of terminology, a positive screening for a SDoH domain (ie, “yes” for housing insecurity) in this study is referred to as an adverse SDoH.^11^ Patients who reported at least one adverse SDoH were provided with a printed referral guide comprised of a list of community resources frequently used by the ED social work team. We then assessed patients’ perceived impact of COVID-19 on their SDoH. All screening data were recorded both in REDCap electronic data capture tools hosted at Boston University Medical Center and in the electronic health record. Two to three weeks after the screening, RAs completed follow-up surveys by telephone to identify patients’ preferred methods of resource referral and any barriers to resource connection and utilization. A maximum of five attempts for contact (phone call or text) were made before a patient was deemed lost to follow-up.

### Outcomes

The primary outcome featured the prevalence and distribution of eight SDoH of ED urgent care patients during the COVID-19 pandemic. The secondary outcomes included feasibility of SDoH screening and referral in ED urgent care, assessment of patient-perceived impact of the COVID-19 pandemic on SDoH, and the preferred methods of resource referral.

### Analysis

Descriptive statistics were obtained using SAS v 9.4 (SAS Institute Inc, Cary, NC) software for patient age, gender, race, ethnicity, preferred language, and ZIP code. We then analyzed prevalence of each SDoH was by demographic using chi-square and Fisher exact tests, and we analyzed the impact of COVID-19 on reporting one adverse SDoH using logistic regression.

## RESULTS

### Characteristics of Study Subjects

Of the total estimated 2018 ED urgent care patients during the times of screening over the course of 13 weeks, 418 patients (20.7%) were presented with a screener via the convenience sample method. Of those presented with a screener, 414 (99.0%) patients completed the screening. The participants were predominantly male (229, 55.3%), averaging 43 years old. The participants self-identified as Black non-Hispanic (58.7%), Hispanic (19.8%), White (16.2%), and other (Table 1). Preferred languages of the participants included English (89.1%), Spanish (6.0%), Haitian Creole (2.2%), and other. The population captured in this study, when compared to that of the general patient population seen in the adult ED of this hospital, was found to have differences including more Black non-Hispanic (this study 58.7% vs adult ED 41.0%), less Hispanic (19.8% vs 26.4%), as well as more English-speaking (89.1 vs 71%), among others.

### Main Results

Of those screened, the majority (71.5%) reported at least one adverse SDoH. The most commonly reported SDoH was interest in further education (38.7%), followed by food insecurity (35.3%), and unemployment (31.0%). Housing insecurity was reported by 21.0%. Of those who reported at least one adverse SDoH, 83.5% requested help and further resources during the visit.

As for the COVID-19-related results, a total of 236 (57.0%) patients reported that their SDoH were negatively impacted by the pandemic. Within each category of SDoH, caregiving for the elderly and children was most likely to have been negatively impacted by the pandemic (86%), followed by employment (84%) and paying for medication (84%) (Table 2).

As for the feasibility of the SDoH screening, the reported time taken from recruiting a patient to completing the screening ranged from 5–10 minutes. Almost all patients (414/418, 99%) completed the screening when approached by trained RAs. Of the 234 attempted follow-up calls, 156 (67%) patients were successfully reached during follow-up phone calls. Of those, 36 (23.1%) reported attempting to connect with a resource, with most attempts made for housing (11.0%) and food (7.7%). Reasons for not contacting the provided resources included lack of time (37.8%) and forgetting to do so (26.3%). Patients preferred resource guides to be printed (34.0%), sent via text message to their mobile devices (25.6%), and explained in person by a resource navigator (23.1%).

## DISCUSSION

This study supports the hypothesis that many patients of this urban, safety-net hospital’s ED urgent care have notable adverse SDoH, thereby signifying a much-needed continued effort in implementing universal SDoH screening in ED urgent care settings. More than half of all screened ED urgent care patients endorsed COVID-19’s impact on their SDoH, shedding light on the tangible toll the pandemic has taken on this patient population. When a dedicated, trained staff member approaches patients, the SDoH screening process was shown to be feasible. However, the follow-up survey reveals a clear discrepancy between referral guide provision and patients’ likelihood to connect with a resource.

To address this gap, departments should increase availability of printed or electronic SDoH resource guides and consider engaging resource navigators with interpreter services both during and after ED visits. Next steps in this endeavor include optimizing the time point of screening (triage, waiting room, patient room), more training of RAs to minimize bias and improve rapport, and deciding who should perform the screening for practicality and efficiency in the ED. Another next step is to consider digital screening tools (tablet, computer kiosk), as prior studies have shown feasibility and acceptability of digital screening for social risk.^12^,^13^

## LIMITATIONS

Limitations of this study include the lack of randomization and potential bias in the selection of convenience samples in ED urgent care between the hours of 8 am–4 pm. Given that patients who frequently visit the general adult ED of this safety-net hospital during evening hours anecdotally have higher rates of housing and food insecurity, and different illness acuity, the fact that they were not included in the urgent care study means we may have underestimated the prevalence of certain SDoH; it may also explain the difference in race, ethnicity and preferred languages seen in this cohort when compared to the general adult ED population. This limits the applicability of our findings to the general population.

## CONCLUSION

Many ED urgent care patients in this study reported at least one adverse social determinant of health, the majority of which were exacerbated by the COVID-19 pandemic. Hospitals should factor in the findings of this study as they prepare for the negative social impacts from the COVID-19 pandemic, highlighting the need to allocate more resources to standardize and expand SDoH screening in EDs and to increase availability of printed or electronic SDoH resource guides, resource navigators, and interpreters both during and after ED visits.

## Figures and Tables

**Table 1:** Demographics of the emergency department urgent care patients who participated in social determinants of health screening.

Demographic Information	Adverse SDoH, n (%)	No Adverse SDoH, n (%)
Age	n = 296	n = 118
18–35	106 (35.81)	39 (33.05)
36–50	97 (32.77)	36 (30.51)
51–65	80 (27.03)	37 (31.36)
66–75	12 (4.05)	5 (4.24)
75–80	1 (0.34)	1 (0.85)
Gender		
Male	167 (56.4)	62 (52.4)
Female	129 (43.6)	56 (47.5)
Race and ethnicity		
Black Non-Hispanic	174 (58.78)	69 (58.47)
Hispanic/Latino	56 (18.92)	26 (22.03)
White Non-Hispanic	51 (17.23)	16 (13.56)
Unknown	8 (2.7)	2 (1.69)
Asian	4 (1.35)	4 (3.39)
Other	3 (1.01)	1 (0.85)
Preferred Language		
English	268 (90.54)	101 (85.59)
Spanish	16 (5.41)	9 (7.63)
Haitian Creole	7 (2.36)	2 (1.69)
Portuguese	2 (0.68)	0 (0)
Other	2 (0.68)	3 (2.54)
Cape Verdean Creole	1 (0.34)	3 (2.54)

*SDoH*, social determinants of health.

**Table 2:** Effect of COVID-19 pandemic on each social determinant of health domain.

Social Determinant of Health Domain (SDoH)	Adverse SDoH, n (%)	No Adverse SDoH, n (%)	*P*-value
Education	n = 160	n = 254	
*COVID-19 Impacted*	111 (69.38)	125 (49.21)	<.0001
*COVID-19 not impacted*	49 (30.63)	129 (50.79)	
Food	n = 146	n = 268	
*COVID-19 impacted*	117 (80.14)	119 (44.40)	<.0001
*COVID-19 not impacted*	29 (19.86)	149 (55.60)	
Employment	n = 128	n = 286	
*COVID-19 impacted*	107 (83.59)	129 (45.10)	<.0001
*COVID-19 not impacted*	21 (16.41)	157 (54.90)	
Utilities	n = 96	n = 318	
*COVID-19 impacted*	77 (80.21)	159 (50.00)	<.0001
*COVID-19 not impacted*	19 (19.79)	159 (50.00)	
Living Situation	n = 85	n = 329	
*COVID-19 impacted*	69 (81.18)	167 (50.76)	<.0001
*COVID-19 not impacted*	16 (18.82)	162 (49.24)	
Transportation	n = 83	n = 331	
*COVID-19 impacted*	65 (78.31)	171 (51.66)	<.0001
*COVID-19 not impacted*	18 (21.69)	160 (48.34)	
Medicines	n = 74	n = 340	
*COVID-19 impacted*	62 (83.78)	174 (51.18)	<.0001
*COVID-19 not impacted*	12 (16.22)	166 (48.82)	
Caregiving	n = 37	n = 377	
*COVID-19 impacted*	32 (86.49)	204 (54.11)	<.0001
*COVID-19 not Impacted*	5 (13.51)	173 (45.89)	

COVID-19, coronavirus 2019.
